# Effect of orally-administrated thymoquinone during pregnancy on litter size, pentylenetetrazol-induced seizure, and body weight in rat offspring

**DOI:** 10.22038/ijbms.2020.47479.10930

**Published:** 2021-01

**Authors:** Amin Abdollahzade Fard, Ehsan Saboory, Yaghob Tahmazi, Yousef Rasmi, Sina Dindarian, Negin Parsamanesh

**Affiliations:** 1 Department of Physiology, Faculty of Medicine, Urmia University of Medical Sciences, Urmia, Iran; 2 Zanjan Metabolic Diseases Research Center, Zanjan University of Medical Sciences, Zanjan, Iran; 3 Cellular and Molecular Research Center, Urmia University of Medical Sciences, Urmia, Iran; 4 Student Research Committee, Urmia University of Medical Sciences, Urmia, Iran

**Keywords:** Nigella sativa, Pentylenetetrazol, Pregnancy outcomes, Seizure, Thymoquinone

## Abstract

**Objective(s):**

This study aimed to assess the impact of orally-administrated thymoquinone (TQ) during pregnancy on litter size, pentylenetetrazol-induced seizure, and body weight in rat offspring.

**Materials and Methods:**

In this experimental study, 64 pregnant rats were divided into groups according to the doses of TQ (0,10, 40, and 80 mg/kg) and gestational week (GW2 and GW3) of TQ administration. After parturition, the pups were counted, weighed, and assessed for pentylenetetrazol (PTZ)-induced seizure on postnatal days 14 (P14) and 21 (P21).

**Results:**

In GW2 treated rats, TQ 40 mg/kg decreased seizure stages compared with control only on P14 while seizure duration significantly decreased on P14 and P21. On P14, 40 mg/kg TQ increased latency to the first seizure but decreased it on P21. In addition, 40 mg/kg dose decreased body weight (BW) on P1, P14, and P21 compared with 10 mg/kg dose and control groups. The dose of 80 mg/kg led to a complete pregnancy loss. In GW3 treated rats, only 10 mg/kg TQ decreased the seizure stages on P14 and P21. None of the doses had a significant effect on seizure duration and latency. TQ 40 and 80 mg/kg led to a low birth weight while increased BW on P14 and P21. A 50% decrease in litter size was observed in 80 mg/kg treated rats.

**Conclusion:**

Prenatal TQ may have anticonvulsant effects. The effects of TQ on BW of offspring depend on its dose and administration time. Also, a high dose of TQ at GW2 can be severely toxic for pregnancy.

## Introduction

Early life events can affect offspring neurodevelopment including seizure susceptibility in both short-term and long-term (1-3). Human and animal studies have demonstrated that the nutrition of the mother during pregnancy is an important environmental factor that plays a role in offspring neurodevelopment and mental health (4, 5). This nutrition can affect the mental health of offspring in both negative and positive ways. An unhealthy diet and harmful exposures during pregnancy can lead to neurodevelopmental problems (6), while a prenatal healthy diet and lifestyle may benefit neurodevelopment (7). 

For centuries, medicinal plants have been studied and used for the treatment of diseases including neurological disorders such as seizure and epilepsy. For instance, *Nigella sativa* (*N.* *sativa*), which belongs to the Ranunculaceae family and is known as the black seed, has a wide range of therapeutic and biological potentials (8). This plant has been extensively used as a remedy for several diseases in traditional medicine (9). Thymoquinone (TQ) is the core component of *N.* *sativa* and it is responsible for most of its effects. It has been found that TQ may have analgesic and anti-inflammatory potentials and protect the body against chemical carcinogens and inhibit membrane lipid peroxidation (10-12). Also, it has been reported that TQ and its other analogs such as para-benzoquinone, 2-methyl-para-benzoquinone, and 2-isopropyl-para-benzoquinone have anticonvulsant effects and suppress seizure (13-15). Herbal products have been a major source of remedies and are extensively used worldwide. Although their side effects are less recurrent than those of synthetic drugs, the notion that natural products are fully safe and without any adverse effects is incorrect (16). The potential effect and/or toxicity of drugs in pregnancy should be tested before their use in pregnant mothers. Thus, based on a broad range of biological effects of *Nigella* *sativa* oil (*N. sativa *oil) and its core component TQ, its use is possible in women during pregnancy. Some women continue to use opiates when they are pregnant. The prevalence of opiate dependence during pregnancy has been increased from 0.17% (1998) to 0.39% (2011) for an increase of 127% in united states (17). Realizing the extensive attention given to *N.* *sativa* and TQ in opioid withdrawal studies, TQ has been suggested to be used as a new potential supplement for methadone maintaining therapy (MMT) towards optimizing the output of this program. The basis of this suggestion is believed to be mediated by calcium channel that is in line with the properties of TQ as calcium channel blocker (18). 

Another issue of TQ use in pregnancy might be control of hypertension. High blood pressure in pregnancy can be due to essential- or pregnancy-induced hypertension. It has been reported that TQ decreases arterial blood pressure, heart rate, coronary artery disease, and peripheral vascular disease (19). Hypertensive patients need to take drugs to control the hypertension and prevent or minimize the complications. Some of these drugs may have adverse side effects, particularly, in pregnancy. Therefore, it is sensible to examine precisely, complimentary remedies that are more effective and with minimal adverse effects. In a systematic review, the findings of 11 studies on humans exploring the antihypertensive effects of *N.* *sativa* treatment have been reported (20). 

 Therefore, the potential adverse effects of TQ are required to be tested before its clinical applications in animal studies. Since the anticonvulsant effects of TQ exposure during pregnancy have not been investigated in offspring, this study aimed to assess the impact of orally administrated TQ during pregnancy on litter size, Pentylenetetrazol (PTZ)-induced seizure, and body weight in rat offspring.

## Materials and Methods

***Ethical approval***

All procedures were reviewed and approved by the Ethics Committee at Urmia University of Medical Sciences, Urmia, Iran with a registration number of 94-01-57-1952 in June 2018. Also, the procedures were conducted in accordance with the 1964 Helsinki Declaration and its later amendments, as well as Principles of Laboratory Animal Care (NIH publication Vol. 25, No. 28 revised 1996).

***Animals and study design***

In this experimental study 64 ten-week-old virgin female Wistar rats (weighing 180-210 g) were included. They were kept in standard conditions including 22±2 °C, *ad libitum* feeding, and 12-hr light/12-hr dark cycle (light on at 7 am). After 2 weeks, each rat was mated with a sexually experienced male rat in a separate cage. The first day of pregnancy was based on vaginal plaque seen after mating. We kept the pregnant rats in groups of four per cage in standard conditions as stated. They were divided into 8 groups each with 8 members as below:

*A) The rats treated at second gestational week (GW2)*

1) Control-GW2: received 1 ml ethanol 25% (v/v) by gavage for seven consecutive days, 2) TQ10-GW2, 3) TQ40-GW2, and 4) TQ80-GW2. The rats in the groups 2, 3, and 4 received 10, 40, and 80 mg/kg TQ dissolved in 1 ml ethanol 25% by gavage for seven consecutive days, respectively.

*B) The rats treated at third gestational week (GW3)*

5) Control-GW3: received 1 ml ethanol 25% (v/v) by gavage for seven consecutive days, 6) TQ10-GW3, 7) TG40-GW3, and 8) TQ80-GW3. The rats in the groups 6, 7, and 8 received 10, 40, and 80 mg/kg TQ dissolved in 1 ml ethanol 25% by gavage for seven consecutive days, respectively. 

Previous studies have revealed that GW2 and GW3 are more sensitive to environmental and social factors to induce changes (including seizure susceptibility) in offspring (3, 21, 22); therefore, GW2 and GW3 were designated for TQ administration in this study. 

The materials used in this study were TQ (purity ≥98%, CAS N° 490-91-5, Cayman, USA), PTZ (purity ≥98%, Sigma Aldrich, Germany), and ethanol (purity ≥99%, Merck, Germany). The solubility of TQ in pure ethanol is 16 mg/ml at room tempreture (22 °C) according to provider company. It is sparingly soluble in aqueous buffers. At first, TQ was dissolved in pure ethanol and then diluted with the distilled water to achive maximum favorable dilution of ethanol to minimize the alcohol consumption as a vehicle. Ethanol 25% (v/v) was the maximum dilution of vehicle that TQ was dissolved at 38 °C.

At gestational day 20, the rats were individually transferred into separate cages under the same conditions. After parturition, the newborn pups were counted and culled to 8, weighed, and divided into groups of 16 based on their mothers’ grouping. One male and one female pup per dam were selected in each identified group (n=16, 8×2=6). Except for group TQ80-GW2 that had no parturition, all pregnant rats in this group missed fetuses. The day of delivery was considered as the first postnatal day (P1). On P14 and P21, we induced seizures using PTZ (45 mg/kg, IP) for both male and female pups. Then, the rats were transferred to a glass chamber (30×30×30 cm^3^) to observe their epileptic behaviors for 60 min. The level of seizure activity was determined according to the five-stage criteria of Racine *et al*. (23) (Table 1). We also recorded seizure stage, seizure duration, latency to the first seizure, latency to tonic-clonic (TC) seizure, number, and duration of TC seizure for each rat. Each pup was evaluated for seizure only once. Hence, different pups were examined on P14 and P21. Previous studies have shown that P14 and P21 are appropriate for evaluating seizure in rats (21, 24, 25); thus, these days were chosen in the current study.

***Statistical analysis***

Data are expressed as mean±standard error of mean (SEM). We used the Kolmogorov-Smirnov test to check the distribution of data. All the data related to PTZ-induced seizures did not have a normal distribution. For normally distributed data and those without normal distribution, parametric techniques and Kruskal-Wallis and/or Mann-Whitney U tests were used, respectively. Also, analysis of variance (ANOVA) was used for analyzing mean differences among groups on body weight (BW) and litter size. All analyses were conducted using IBM SPSS Statistics 22 (SPSS Inc., Chicago, IL, USA). Differences with *P*<0.05 were considered statistically significant**. **

## Results

At first, data related to epileptic behaviors were compared between male and female pups. Since there was no significant difference between the two sexes, data of male and female pups were combined and analyzed together.

***Treatment at GW2***

All rats in the GW2 treatment stage had a successful pregnancy and had a litter size compatible with the control group (11.12±0.61) except the rats in the TQ80-GW2 group, which had no parturition. All rats in this group lost their pregnancy with significant BW loss from gestational day 9 to 14 (one day after TQ administration). Then, they reached pre-pregnancy BW until the end of the study. 

***Effects of TQ on seizure in offspring***

**Figure 1:**
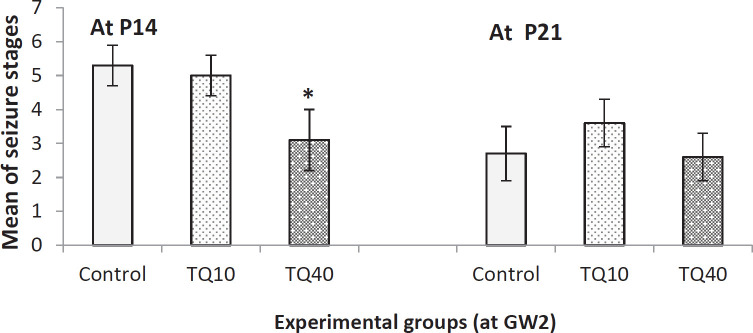
Evaluating seizure stages of PTZ-induced seizure in 14- and 21-day old rat pups which their mothers received TQ at 2^nd^ gestational week (GW2); * indicates *P*<0.05 VS control group at P14

Figure 1 shows seizure stages in 14-day and 21-day old pups that their mother received 10 and 40 mg/kg TQ at GW2. As shown in this Figure, on P14, 40 mg/kg of TQ led to decreased seizure stages compared with control and TQ10 groups. No significant decrease was observed in seizure stages between control and TQ-10 groups. Also, on P21, none of the TQ doses decreased the seizure stages compared with the control group (Kruskal-Wallis and/or Mann-Whitney U tests). 

**Figure 2:**
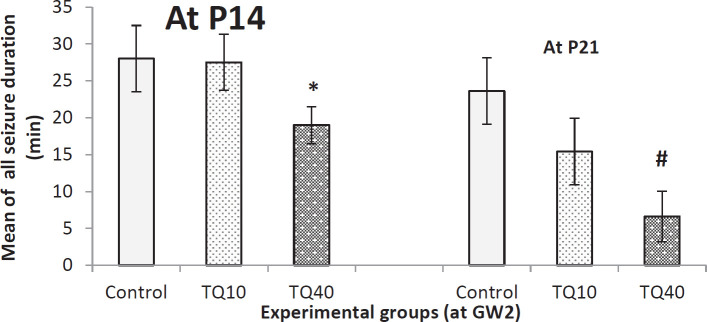
Seizure duration in 14- and 21-day old rat pups which their dam received TQ (10 and 40 mg/kg) at 2^nd^ gestational week (GW2); *indicates *P*<0.05 Vs control and TQ10 at P14; # indicates *P*=0.04 Vs control group

Assessing the seizure duration in rat offspring that their mothers received TQ at GW2 is shown in Figure 2. According to this Figure, at both days, only 40 mg/kg significantly decreased seizure duration compared with the control group. Seizure duration was calculated as the summed duration of all epileptic behaviors from stages 1 to 5. 

**Figure 3:**
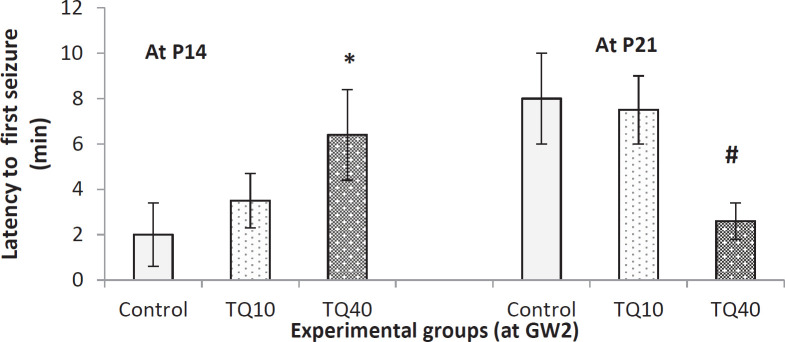
Latency of first seizure in 14- and 21-day old rat pups which their dam received TQ (10 and 40 mg/kg) at 2^nd^ gestational week (GW2); * indicates *P*<0.01 VS control at P14; # indicates *P*<0.03 VS control at P21

Figure 3 demonstrates the seizure latency on P14 and P21 that prenatally (at GW2) exposed to TQ (10 and 40 mg/kg). On P14, 40 mg/kg dose of TQ significantly increased latency to the first seizure compared with the control group. In comparison, it decreased latency to the first seizure on P21 (Kruskal-Wallis and Mann-Whitney). 

**Figure 4:**
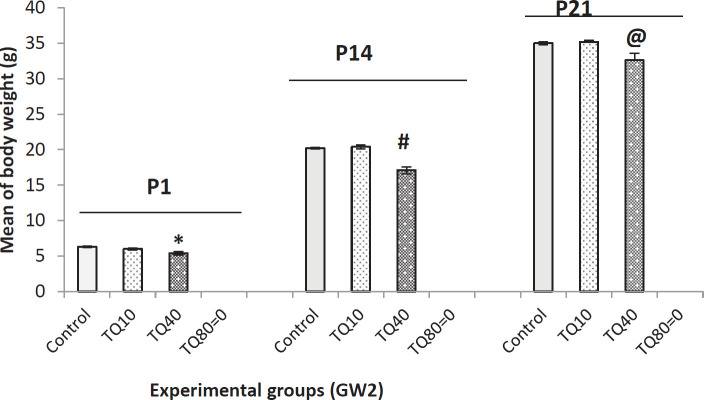
Body weight at P1, P14, and P21 in rat offspring which their mothers received TQ at 2^nd^ gestational week (GW2); * indicates* P*=0.003 VS control at P1, # indicates *P*=0.001 VS control and TQ10 at P14, and @ indicates *P*=0.009 VS control and TQ10 at P21 (ANOVA and Tukey)

Figure 4 shows that the administration of TQ (only 40 mg/kg) at GW2 had a negative effect on body weights of offspring on P1, P14, and P21 compared with TQ10 and control groups. 

***Treatment at GW3***

Unlike the pregnant rats treated with 80 mg/kg TQ at GW2 (with no parturition), the rats in the TQ80-GW3 group had a successful pregnancy with the same treatment. Eight from ten pregnant rats had a full-term pregnancy and gave birth to offspring (two rats lost pregnancy and were replaced later). An important finding was a diminished litter size in this group that was significantly different from other groups [control= 11.37±0.65, TQ10=11.88±0.91, TQ40=12.13±0.69, and TQ80=5.75±0.45; one-way ANOVA, F (3, 31)=18.93, *P*<0.001]. The litter size in other groups was compatible. 

**Figure 5:**
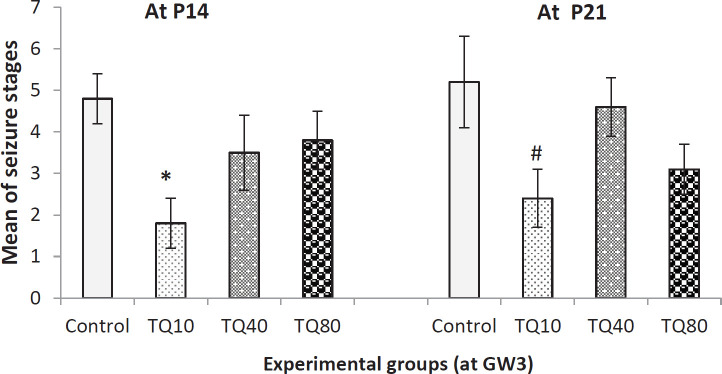
Seizure stages in 14- and 21-day old rat offspring that their mothers received TQ at 3^rd ^gestational week (GW3); * indicates *P*<0.01 VS control at p14 and # indicates *P*<0.05 VS control at P21

As presented in Figure 5, on P14, only 10 mg/kg TQ significantly decreased the seizure stages but, at P21, both 10 and 80 mg/kg of TQ decreased the seizure stages compared with relative control groups (Kruskal-Wallis and Mann-Whitney). 

**Figure 6:**
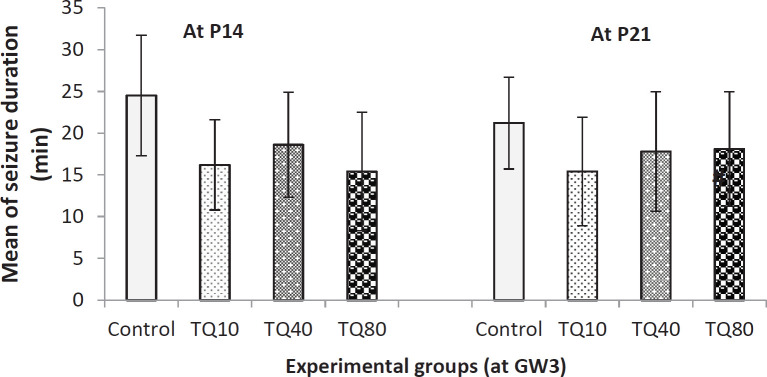
Seizure duration in 14- and 21-day old rat offspring that their mothers received TQ at 3^rd^ gestational week (GW3); there is no significant difference between groups

Also, Figure 6 shows that none of the doses had a significant effect on seizure duration in comparison with the control group; however, there was a non-significant decrease in TQ treated groups. Also, there was no significant difference among groups in terms of latency to the first seizure. But, a non-significant increase was observed in the offspring of TQ treated rats compared with the control group at both P14 and P21.

**Figure 7:**
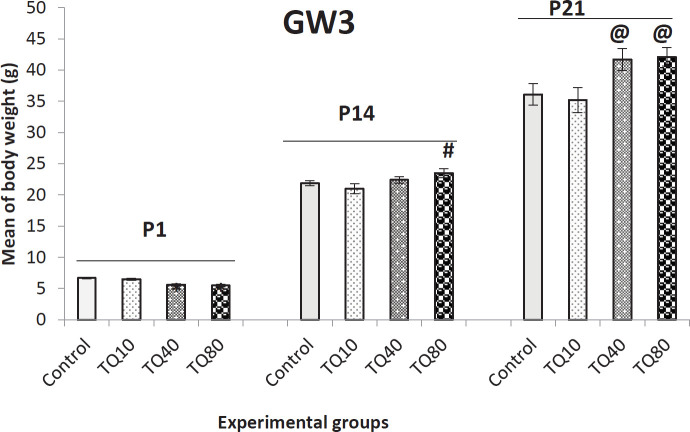
Body weight of 1-, 14-, and 21-day old rat offspring that their dams received TQ at 3^rd^ gestational week (GW3); * indicates* P*<0.001 VS control and TQ10 at P1, # indicates *P*<0.01 VS control and TQ10 at P14, @ shows* P*< 0.01 VS control and TQ10 at P21 (One-way ANOVA and Tukey)

Finally, Figure7 shows the effect of exposure to TQ at GW3 on offspring BW. The pups of dams that received 40 and 80 mg/kg of TQ had higher BWs than offspring in control and TQ10 groups on P14 and P21; meanwhile, these pups had low birth weight (P1) compared to control and TQ10 groups. 

***PTZ- induced epileptic behaviors***

We induced seizure on P14 and P21 by PTZ. Immediately after PTZ injection (40 mg/kg, IP), the behaviors of rats were inspected. The most frequent behavior among the rats was the sudden extension of posterior limbs. Also, clonus of anterior limbs was observed in some rats. Some rats entered stage 4, which involved severe seizure with kangaroo posture or violent convulsion. Some other rats entered stage 5 that consists of continuous tonic-clonic seizure and loss of consciousness. The tonic-clonic seizure was not observed in some rats while, in some rats, it occurred for more than once. 

**Table 1:** Racine criteria for evaluation and staging of seizure (23)

**Activity**	**Stage**
Normal	0
Immobilization, sniffing, closed eyes, ear movement, face clonus	1
Head nodding, facial and forelimb clonus (short myoclonic jerk)	2
Continuous myoclonic jerk, tail rigidity	3
Generalized limbic seizures with kangaroo posture or violent convulsion	4
Continuous generalized seizures (tonic or tonic-clonic convulsions)	5

PTZ: pentylenetetrazol; TQ: thymoquinone

TQ: thymoquinone

TQ: thymoquinone

TQ: thymoquinone

TQ: thymoquinone

TQ: thymoquinone

TQ: thymoquinone

## Discussion

In the current study, we administered TQ during pregnancy to rats and then assessed the effects of TQ on litter size, PTZ-induced seizure, and BW in rat offspring. The results of our study demonstrated that the administration of TQ during pregnancy may generally decrease the seizure stages and increase the latency to the first seizure in rat offspring (inhibitory effect). Also, the pups showed a low birth weight. Then, they gained more weight from P14 until P21. The effects of TQ (on all studied parameters) were dose-dependent and time-specific. TQ 80 mg/kg at GW2 was completely toxic for pregnancy; therefore, all rats in this group (TQ80-GW2) lost the pregnancy. 

In the current study, we used PTZ to induce seizures. PTZ is validly used as a seizure inducer in rodents and it has been used in previous studies (26, 27). Anticonvulsant effects of TQ have also been evaluated in some other epilepsy models. In a study conducted by Ezz *et al.* (28), they evaluated the anticonvulsant effects of *N.* *sativa *oil and curcumin on pilocarpine-induced epilepsy and compared their effects with valproate. They concluded that the treatment with *N.* *sativa*O, curcumin, or valproate could ameliorate the changes induced by pilocarpine. However, the adverse effects of *Nigella* *sativa* oil and curcumin were less than those of valproate. The anticonvulsant effects of curcumin and *Nigella* *sativa* oil might be due to their anti-oxidant and anti-inflammatory effects that have been also reported in some other studies (29, 30). In a study by Noor *et al.* (31), the anticonvulsant effects of NOS and curcumin were compared with those of valproate in the pilocarpine model of epilepsy. Unlike our study, they observed that valproate and curcumin ameliorated the pilocarpine-induced seizure, but the treatment with *N.* *sativa *oil failed to ameliorate the seizure. Additionally, Beyazcicek *et al*. (32) evaluated the antiepileptic effects of TQ on penicillin-induced epileptiform activity in rats. They observed that TQ increased the latency to the first seizure and decreased the frequency and amplitude of the seizure. Also, Hosseinzadeh *et al.* (14) investigated the anticonvulsant effects of TQ in PTZ-induced and maximal electroshock (MES)-induced epilepsy models. According to their results, TQ decreases the onset and the duration of the seizure in the PTZ model but it does not decrease the onset and duration in MES-induced seizure. However, the protection of TQ against mortality was similar in both models. The results of the current study are consistent with the above-mentioned studies. Nevertheless, there is a big difference in methodology among our study with others: we used TQ during gestation and epileptic behaviors were studied on P14 and P21. In comparison, in all the studies mentioned here, TQ and/or *N.* *sativa *oil was directly used on subjects and epileptic behaviors were studied immediately. Many studies have assessed the mechanism through which TQ affects seizure characteristics. A study by Hosseinzadeh *et al.* (15) demonstrated that intracerebroventricular injection of TQ in the PTZ-induced seizure model decreases the duration of seizure and prolongs its onset. They suggested that the anticonvulsant effect of TQ may be due to an opioid receptor-mediated increase in GABAergic tone. Also, Ullah *et al.* (33) stated that TQ prolongs the onset of PTZ-induced seizure and decreases the high-grade seizures by reversing the PTZ-induced changes in expression of GABA_B1_ receptor, calcium/calmodulin-dependent protein kinase II (CaMKII), and cAMP response element-binding protein (CREB). In other words, PTZ decreases the expression of GABA_B1 _receptor and CaMKII and inhibits the phosphorylation of CREB but TQ reverses these changes caused by PTZ. Moreover, Shao *et al.* (34) studied the protective effects of TQ in a model of status epilepticus (SE) and investigated its mechanism. SE defined as epileptic seizures lasting for more than 5 minutes. Several medications including benzodiazepines, sodium valproate, phenytoin, and phenobarbitone have been suggested for the treatment of SE (35-37). In this regard, Shao *et al.* concluded that TQ may also be used as a therapeutic drug in the treatment of SE due to its anti-inflammatory effects through decreasing the expression of cyclooxygenase-2 (COX-2), tumor necrosis factor-alpha (TNF-a), and nuclear factor-kB (NF-kB) in the hippocampus (34). Also, Arafa *et al.* (38) investigated the anticonvulsant effects of TQ through its effects on amino acid neurotransmitters. They observed that in the rats treated with PTZ, the brain levels of aspartate and glutamate are decreased and the concentrations of GABA and glycine are elevated. The oral administration of *N.* *sativa *oil increased aspartate and glutamate contents and decreased GABA and glycine levels. As GABA and glycine are known as central inhibitory amino acids and glutamate and aspartate are observed to have excitatory roles (39), it can be concluded that treatment with PTZ may induce imbalance between inhibitory and excitatory amino acids leading to initiation of seizure; nevertheless, it seems that administration of TQ ameliorates the imbalance between inhibitory and excitatory amino acids. 

Prenatal brain development is characterized by rapid structural and functional changes. Many environmental factors such as nutrition, toxic substances, and stressors affect this development (40, 41). Unfortunately, there is no study available on effect of prenatal exposure to TQ on brain structure and functions. Few studies have reported neuroprotective effects of *N.* *sativa* and/or TQ during neonatal and pregestational period. In a study, the effects of feeding by the hydro-alcoholic extract of *N.* *sativa* during neonatal and juvenile growth on learning and memory of rats were investigated. Pregnant rats after delivery, received different concentration of the extract (100, 200, and 400 mg/kg) in drinking water from P1 to P56. Then, male offspring were tested in the Morris water maze. Also, the brains were removed and malondialdehyde (MDA) concentrations were determined. The result showed that feeding of *N.* *sativa* during neonatal and juvenile growth improved learning and memory of rats; the authors concluded that the effects might be due to the anti-oxidant properties because treatment with *N.* *sativa* decreased the MDA content in hippocampal tissues (42). In another study, effect of pre-gestational feeding with TQ on PTZ-induced seizure in rat offspring was investigated; female Wistar rats were fed with TQ for a week. Then, the female rats were mated with male rats. After delivery at P14 and P21, the pups were subjected to PTZ-induced seizure. It was concluded that feeding with TQ before pregnancy suppressed generalized PTZ-induced seizure in rat offspring (43). 

Another finding of the current study was on pregnancy outcomes and BW of offspring. As stated in the result section, TQ 80 mg/kg at GW2 was totally toxic for pregnancy; therefore, all the rats lost their pregnancy. The effect of a single intraperitoneal administration of TQ on the pregnant rat and embryo-fetal development was investigated in a study. Pregnant rats received 15, 35, and 50 mg/kg of TQ on gestational days 11 or 14 (G11 and G14), and sacrificed on G18. Based on the obtained results, the rats treated with 50 mg/kg on G11 showed a significant decrease in BW and complete loss of pregnancy. But, in the rats treated with the same dose, on G14, 46.2% of implants were resorbed and the viable fetuses showed no TQ-related malformations. Meanwhile, with a lower dose (35 mg/kg), toxicities were detected only when TQ was given on G11. In addition, no harmful effect was observed for dams and fetuses with a dose of 15 mg/kg on G11 and G14. Then, it was concluded that TQ, at doses of 50 and 35 mg/kg, might have a harmful effect on embryonic development during the GW2 of rat pregnancy (44). The results of this study are consistent with our findings where all rats in the TQ80-GW2 group lost pregnancy while 20% (2 from 10) rats in the TQ80-GW3 group had such an experience. Moreover, our result showed that the litter size in the TQ80-GW3 group significantly was decreased compared to other groups including control rats. Since we administered TQ in TW3, the decrease in litter size might be due to the resorption of about 50% of fetuses in these rats because of high dose TQ consumption. However, we did not find any more relevant studies to further discuss this finding. Lower BW in offspring of the TQ40-GW2 group on P1, P14, and P21 might be linked to the fetal toxicity reported above. Also, lower birth weight in TQ40-GW3 and TQ80-GW3 potentially has the same mechanism. Besides, we detected increased BW in offspring of TQ40- and TQ80-GW3 groups on P14 and P21. In a study, the effects of *N.* *sativa *oil on blood homeostasis and BW were investigated. Adult rats were treated with *N.* *sativa *oil (1 ml/kg, daily, oral) for 12 weeks. A significant decrease in the BW was observed in *N.* *sativa *oil treated rats compared to the control group (45). In a meta-analysis study, 13 randomized clinical trials, including 875 subjects (64% males), were included in the investigation; the result showed that *N.* *sativa* supplementation significantly reduced BW and body mass index (46). Our finding is against these studies because we showed that TQ during gestation led to an increase in offspring BW on P14 and P21. However, there are remarkable differences in methodology of these studies in comparison to ours; they used *N.* *sativa* and *N.* *sativa *oil directly on subjects but we used TQ on mothers and BW was monitored in offspring at least three weeks later on P14 and P21. Nevertheless, we did not find more relevant studies to further discuss this issue. 

## Conclusion

The results of our study show that the administration of TQ during pregnancy may have anticonvulsant effects. The effects of TQ on BW of offspring depend on its dose and the time (gestational week) of administration. High dose TQ (orally, 80 mg/kg), particularly at GW2, might have adverse pregnancy outcomes such as complete pregnancy loss. To the best of our knowledge, the current study is the first study assessing the anticonvulsant effects of prenatal administration of TQ on seizure in offspring. So, we recommend conducting further studies with molecular and biochemical analyses to reach a comprehensive conclusion. 
